# COVID-19 Syndemic, Government, and Impact on Mental Health: A Brazilian Reality

**DOI:** 10.3389/fpsyt.2021.671449

**Published:** 2021-08-06

**Authors:** Estelita Lima Cândido, Jucier Gonçalves Júnior

**Affiliations:** ^1^Postgraduate Program in Health Sciences, School of Medicine and Postgraduate Program in Sustainable Regional Development (PRODER), Universidade Federal do Cariri (UFCA), Barbalha, Brazil; ^2^Division of Rheumathology, Department of Internal Medicine, Universidade de São Paulo (USP), São Paulo, Brazil

**Keywords:** COVID-19, disaster behavioral, mental health, public health, syndemic, panic

## Introduction

Pandemics are natural disasters known as epidemics that spread rapidly across countries and affect large numbers of people worldwide. They have consequences at the micro- and macrosystemic levels and impose new rules, social habits, and mobilizations of different natures on the affected population (1). During these historical moments of stress, there is a significant amount of psychological suffering, increasing the incidence of fear, insecurity, anxiety disorders, depression, and suicide (2).

In some countries, such as Brazil and the USA (3), politicians saw an opportunity for individual or party promotion in the pandemic and began to act irresponsibly, which diverged from what the moment demanded of their position. They managed to challenge the credibility of science and organizations like the World Health Organization (WHO), which play a decisive role in increasing the humanity's expectation and quality of life. Therefore, instead of maintaining the collective unit to face the disease, the population was divided. Part of the people maintained their reasoning, collaborating with the recommendations of the authorities, yet another only corroborated and followed the party guidelines (4).

In Brazil, dozens of people, including businessmen, health officers, governors, and mayors, are being investigated or responding to overpricing processes, fraud, and other misuse forms of resources that culminated in the collapse of the hospital network and high number of deaths. The most recent case occurred in Manaus, capital of the Brazilian state of Amazonas, where patients were not only accumulated in the corridors due to lack of beds but also lacked oxygen. Horror scenes were broadcast by health professionals and residents asking for help, and family members of sick individuals carrying oxygen cylinders on their shoulders in an attempt to save their suffocating loved ones (5, 6).

This political and social instability scenario promotes fear, anguish, social instability, and worsening of existing psychiatric diseases. According to the WHO, Brazil is the second country with the highest number of people with depression in the Americas (5.8%) and with the highest prevalence of anxiety disorders (9.3%) (7).

When a country's population already presents risk factors, such as low socioeconomic indexes and high incidence of psychiatric diseases, poor management during the pandemic can be a determining factor for the population's mental illness. Thus, the objective was to discuss the Brazilian challenges to promote mental health during the COVID-19 pandemic and the Brazilian Government's performance in this context.

## Discussion

Brazil has demographic and economic characteristics that have been consistently pointed out as the backdrop to the increased incidence and persistence of mental disorders in its general population (8).

The first major Brazilian challenge has been to live with the deepening of social inequalities in the midst of the economic crisis provided by COVID-19. According to data from the Brazilian Institute of Geography and Statistics (IBGE) until November 2019, shortly before the pandemic broke out, the income of the wealthiest 10% was 13 times higher than that of the poorest 40% in Brazil (9). A systematic review of studies conducted in low- and middle-income countries reported that more than 70% of the 115 reviewed papers showed an association between different levels of poverty and common mental disorders (10). However, in March 2020, the Brazilian Government issued the provisional measure 927, which provided in its article 18, among other prerogatives, the suspension of employment contracts for up to 4 months, without wage. In addition, they transferred 1 trillion and 200 billion Brazilian real (189.300.700.000 and 37.860.140.000 US dollars, respectively) to the central bank, which has not contributed to any effective measure to combat the pandemic (11).

The second challenge was to put in place public health measures to contain the virus properly. This led to the health crisis perpetuation and the collective emergency state and, therefore, worsened the exposed population's mental health. In Brazil, the measures recommended by international health agencies have been ignored and discredited by the current president on several occasions (12). Drugs without scientific proof were promoted: hydroxychloroquine (13), azithromycin, and ivermectin. In addition, a popular agenda promoting events and agglomerations throughout the country was also maintained (14), and there was also discouragement from wearing masks (15) and disobedience to social isolation (5). The president even promoted a campaign on his social networks entitled “O Brasil não pode parar” (“Brazil cannot stop”) to encourage the return of activities, which was later removed from the air (11). Part of his campaign against the vaccine has also become popular around the world with his picturesque statements. For example: “people may become crocodiles,” after getting the vaccine, or the several times that he discredited the effectiveness of vaccines in interviews for Brazilian open TV, and when he called the vaccine produced by the Chinese company Sinovac “Vaccine Communist” (16).

The third Brazilian challenge was to fight against fake news and the provision of quality information that generate anguish, despair, fear, uncertainty, and confusion among the people (17). This “disinformation pandemic,” whose term was recently nicknamed, is called Infodemia (18), and it is being disseminated more quickly than Sars-CoV-2 itself (19). Doubtful or even false information about factors related to virus transmission, incubation period, geographic reach, number of infected people, and real lethality rate led to insecurity and fear in the population (20). Thus, the lack of knowledge about the disease, as well as the easy access to information, whether of quality or not, through traditional media or social networks, served as a catalyst in the development of panic in people, especially those who are socioeconomically and emotionally vulnerable. If the disease, by itself, causes concern and uncertainty, information disseminated premeditatedly to promote chaos only worsens the situation (17).

The fourth challenge was to deal with a pandemic (COVID-19) and epidemic (mental health) at the same time without human, material, nor infrastructure resources (21). According to data from the Department of Science, Technology, Innovation and Communication of Brazil, investment in the portfolio has been decreasing since 2016. At that time 5.8 billion were earmarked for financing. In 2020, 3.6 billion were offered, and in 2021, there is a government proposal, under evaluation by the Brazilian Congress, for a further cut of 34% (22). In addition, there is a consensus in the literature that, after 2011, there has been a systematic underfunding and bureaucratization of the Brazilian Unified Health System (SUS), especially in the organizations that manage mental health (23). In December 2020, the Brazilian government expressed interest in revoking ordinances issued between 1991 and 2014, which regulated programs for restructuring psychiatric hospital care and patient follow-up teams in de-hospitalization (24).

The fifth challenge was the instability of the Brazilian Department of Health, which is directly responsible for the elaboration of public policies to combat COVID-19, due to the differences between the ministers of health and their teams and the president of the republic. Since the beginning of the pandemic, starting in March 2020, the portfolio has had four ministers in charge of it. The reason for this turnover is mainly due to the disagreement of the president of the republic with the posture of past ministers who supported measures such as social isolation, lockdown, use of masks, no support to the use of medications without scientific proof (e.g., hydroxychloroquine), or overvaluation of the economy/opening of public spaces (defended by the president), despite the increase in the number of deaths/confirmed cases of COVID-19 in Brazil (11, 14). According to Jesus et al. (25), at various times, the president of the republic opted for the strategy of minimizing the pandemic by calling it a “*gripezinha*” [small flu]. The measures enacted to combat the pandemic went against his neoliberal interests (25). The Brazilian management, for this reason, was highlighted throughout the world, being an editorial agenda in The Lancet (26).

## Final Considerations

In more than a year since the beginning of the pandemic, Brazil has had more than 15 million infected people and 441 thousand deaths, with daily and uninterrupted records of deaths per day (26). Currently, there has been no coordinated response at the federal level to combat COVID-19, vaccination has been implemented slowly, and health services are overcrowded, with overworked health professionals and a fragile population.

The mental health of the Brazilian people has never been so complex and unprecedented. From the economic crisis to political instability, from the accentuation of social inequalities in the last 10 years to the deepening of poverty/misery in the last 2 years, several factors led us to believe that there is not only a pandemic in Brazil, but a Syndemic. This term refers to “a set of closely intertwined and mutual enhancing health problems that significantly affect the overall health condition of a population within the context of a perpetuating configuration of noxious social conditions” (27, 28).

Thus, in the coming years, what will be seen in Brazil is an incapacitating epidemic of mental disorders in a poor population, with a despoiled health system and without sufficient social support.

To avoid this scenario, a collective effort by Governments at the municipal, state, and federal level is suggested in a broad vaccination campaign, social isolation measures, the use of masks, and combating disinformation. These measures must be reviewed every 15 days by a pre-established crisis committee and, according to the COVID-19 and non-COVID-19 hospitalization rates in each Brazilian state/region, create social mobilization plans (for trade—opening or closing and health—mobilization of beds). At the same time, it is important to orient resources and train labor, using public universities (federal and state), to manage the most common emergencies/cases in mental health. Finally, investing in telemedicine models during the pandemic, as a damage control policy for the most vulnerable patients.

## Author Contributions

All authors contributed to the design, writing, and final reading of the paper.

## Conflict of Interest

The authors declare that the research was conducted in the absence of any commercial or financial relationships that could be construed as a potential conflict of interest.

## Publisher's Note

All claims expressed in this article are solely those of the authors and do not necessarily represent those of their affiliated organizations, or those of the publisher, the editors and the reviewers. Any product that may be evaluated in this article, or claim that may be made by its manufacturer, is not guaranteed or endorsed by the publisher.
